# A CROSS-CULTURAL EXAMINATION OF SOCIAL PARTICIPATION, FAMILY INTERACTION, AND DEPRESSIVE SYMPTOMS

**DOI:** 10.1093/geroni/igad104.2164

**Published:** 2023-12-21

## Abstract

Social participation and family interaction are core components of later life. Less is known about cross-cultural differences regarding the relationships between social participation, financial support, caregiving, and depressive symptoms among older adults. This study aimed to estimate the association between these factors and depressive symptoms among older adults in Europe and China. We selected individuals aged 60 years and above from the 2017 Harmonized Survey of Health, Ageing, and Retirement in Europe (SHARE) (n = 11,676) and the 2018 Harmonized China Health and Retirement Longitudinal Study (CHARLS) (n = 5,559). Stepwise linear regression was used to analyze the data. Participation in social activities was found to be associated with lower levels of depressive symptoms among both European and Chinese older adults. Among Chinese older adults, providing financial transfers to adult children was associated with lower levels of depressive symptoms, whereas transfers to older parents was not significant. Care for older parents was associated with lower levels of depressive symptoms, while caregiving for children or grandchildren was not significant. Among European older adults, providing financial transfers to others was not significant. Care for grandchildren was associated with lower levels of depressive symptoms, while caring for other family members was associated with higher levels of depressive symptoms. In conclusion, participation in social activities, providing financial transfers to adult children, and caring for older parents have positive effect on psychological well-being of Chinese older adults. For European older adults, social activities and caring for grandchildren prove to be more beneficial.

